# A single session of whole-body cryotherapy boosts maximal cycling performance and enhances vagal drive at rest

**DOI:** 10.1007/s00221-022-06528-y

**Published:** 2022-12-21

**Authors:** Jorge L. Storniolo, Marco Chaulan, Roberto Esposti, Paolo Cavallari

**Affiliations:** 1 Human Physiology Section, Department of Pathophysiology and Transplantation, Università degli Studi di Milano, Milan, Italy; 2 M1 Physio Sport Clinic, Milan, Italy

**Keywords:** WBC, Cycloergometer, All-out exercise, HRV at rest, Autonomic nervous system, Human

## Abstract

Whole-body cryotherapy (WBC) has been reported to maximize physical recovery after exercise and reduce the ensuing muscle damage. In addition, WBC triggers cardiovascular responses leading to an increased vagal drive. Here we tested whether WBC may boost exercise performance as well as post-exercise recovery. Moreover, we compared the effects of WBC and exercise on sympathovagal balance and tested whether these two factors may interact. ECG was recorded in 28 healthy adults who underwent rest, all-out effort on a cycloergometer, 5 min recovery and again rest. After 3–5 days, WBC (3 min exposure to − 150 °C air) was applied and the whole procedure repeated. Total exercise duration was split into the time needed to reach peak power output (t_PEAK_) and the time to exhaustion (t_EXH_). The post-exercise exponential decay of HR was characterized by its delay from exercise cessation (t_DELAY_) and by its time constant (τ_OFF_). Sympathovagal balance was evaluated by measuring HR variability power in the low (LF) and high (HF) frequency bands, both before exercise and after recovery from it. Sympathetic vs. vagal predominance was assessed by the sympathovagal index LFnu. Paired t-tests indicated that WBC increased t_EXH_ and reduced t_DELAY_, speeding up the HR recovery. These results suggest that WBC may be exploited to boost exercise performance by about 12–14%. ANOVA on HR variability confirmed that exercise shifted the sympathovagal balance towards sympathetic predominance, but it also highlighted that WBC enhanced vagal drive at rest, both before exercise and after full recovery, covering ~ 70% of the exercise effect.

## Introduction

Cryotherapy refers to an array of therapeutic applications in which the body is exposed to low temperatures to achieve considerable cooling of the skin and the underlying tissues (Costello et al. 2012a, b, 2014). Some of these treatments expose the whole body to a freezing atmosphere (cryochamber, – 110 to – 150 °C for a few minutes), other exclude the head and neck (criosauna); nevertheless, cryochamber and criosauna treatments are commonly referred to as Whole Body Cryotherapy (WBC). This practice (which is now applied also as a wellness treatment method) was introduced in Japan in 1978, developed in Germany and Poland in the `80 s, and quickly gained increasing popularity, especially in sport medicine, for its effects on post-exercise distress (see Banfi et al. 2010 for a review). Despite the very low air temperature, literature does not report skin lesions after this treatment (Westerlund et al. 2003; see also Piotrowska et al. 2021), also considering that the lowest skin temperature remains in the range 15–20 °C. Neither is WBC associated with risk of hypothermia, as core body temperature shows minimal or no appreciable changes in response to that treatment (Komulainen et al. 2004; Costello et al. 2012a, b; Selfe et al. 2014; Zalewski et al. 2014; Cuttell et al. 2017). Moreover, Hammond et al. (2014) reported that body surface area does not influence the magnitude of skin cooling following WBC.

Scientific evidences that cryotherapy may reduce metabolism and inflammation after injury or exercise have been mainly collected in animal models (for a review, see Kwiecien and McHugh 2021). A finding that has been confirmed in humans by Banfi et al. (2009), who showed that in rugby players WBC significantly lowered creatine kinase and lactate dehydrogenase and decreased pro-inflammatory cytokine/chemokine, while anti-inflammatory cytokine actually increased. WBC can also effectively reduce the perceived symptoms of muscle soreness (Rose et al. 2017) as well as reduce pain and improve recovery after exercise (Knight 1995). Moreover, Wozniak et al. (2007) reported that despite WBC increased the oxidative stress, it triggered protective adaptations against the disturbances in the prooxidant–antioxidant balance induced by physical training. Finally, WBC has been proficiently used in acute injuries (in animals, Kafka et al. 2020), in degenerative disorders (Szpruch and Kikowski 2018) and rheumatic diseases (Guillot et al. 2014).

Interestingly enough, very poor attention has been devoted to the effect of WBC on exercise performance. In this regard, it is important to recall that WBC deeply affects the function of the cardiovascular system. Indeed, rapid cooling of nearly the whole body surface has been shown to induce a potent vasoconstriction of the skin, which is associated with an enhanced central blood flow through big vessels (Zalewski et al. 2013). Consequently, the increased blood volume results in a stimulation of the arterial and cardiac baroreceptors, leading to an increased parasympathetic stimulation of the heart (Zalewski et al. 2014; Hausswirth et al. 2013; Louis et al. 2015). Thus, it could be argued that such parasympathetic stimulation might accelerate the post-exercise heart rate (HR) recovery, which in turn seems to be a critical parameter for controlling physical performance (Coote 2010; Michael et al. 2017). Exposure of almost the whole body to cryogenic temperatures induces a decrease in HR and an increase in stroke volume and stroke index; in contrast, the values of systolic, diastolic, and mean blood pressure, as well as of the cardiac index, do not significantly change (Komulainen et al. 2004; Lubkowska and Szygula 2010; Westerlund et al. 2004). However, some studies have documented a significant increase in systolic and diastolic blood pressure as a form of stress response to WBC (Lubkowska and Suska 2011). Other cooling methods are able to affect the autonomic control of the heart, even eliciting opposite effects. Indeed, facial immersion in cold water (about 4 °C, Kinoshita et al. 2006) results in bradycardia, while the “cold pressor test” in which a hand or a foot is immersed in such water for a few minutes (see Lamotte et al. 2021 for a brief review) induces huge sympathetic drive. Thus, the same cooling localized in different body areas may elicit opposite effects. In this regard, it could be argued that the controversial reports on WBC effects on heart and circulation might stem from a non-homogeneous cooling of the skin when exposed to the freezing atmosphere of the WBC.

On these premises, we were interested in analyzing two aspects. First, we wanted to test whether WBC can affect exercise performance as well as post-exercise recovery. We were also interested to compare the effects of WBC and exercise on sympathovagal balance and know whether these two factors may interact. The modulation of the activity of autonomic fibers innervating the heart can be determined non-invasively by measuring the HR variability (HRV), i.e., the fluctuations in time between two heartbeats, (Malliani 2005; Montano et al. 2009). HRV has been widely acknowledged as an indicator of cardiac sympathetic and parasympathetic control (Task Force of the European Society of Cardiology the North American Society of Pacing Electrophysiology 1996). HRV tests do not require expensive apparatuses, besides reliable monitors capable of beat-by-beat acquisition. Current literature reports that WBC involves the autonomic drive at rest (Louis et al. 2015, 2020; Theurot et al. 2021), but these studies did not address the effect of exercise. Instead, Sautillet et al. (2021) tested the WBC effect by applying it only after exercise, thus leading to a cumulative evaluation of both factors.

Thus, the present paper aims to sunder the effects of WBC and exercise on sympathovagal balance, through a full factorial experimental design, so as to evaluate the possible interaction. In particular, we focused on an all-out cycling exercise against a constant-power load, a choice that stems from two reasons. First, the easiness to set a well-defined limit for identifying the end of exercise; second, to be able to measure the time delay (t_DELAY_) occurring between the end of the exercise and the onset of the following exponential HR decay. We were interested in t_DELAY_ since we previously observed (Storniolo et al. 2020) that this parameter is related to the subject’s aptitude to recover from the sympathetic overdrive induced by an all-out exercise. We hypothesize that WBC would increase the exercise duration and speed-up the HR recovery while, in parallel, it would shift the sympathovagal balance toward vagal predominance. Moreover, our experimental design will allow to compare the effects of WBC and exercise on sympathovagal balance and test whether WBC effect is modulated by exercise or if the two effects simply add up to each other.

## Materials and methods

This study followed an Interrupted Time Series Design with a single session of WBC as “treatment”. Inclusion criteria were to be adults under 40 years, with a Body Mass Index between 18.5 and 30.0, and to be physically active (being involved either in a recreational activity or in amateur sports activity with a maximum of three training sessions per week). Exclusion criteria were any history of neurological or cardiovascular diseases and intake of drugs acting on the central nervous system or cardiovascular system. Twenty-eight participants satisfied the above criteria and were enrolled into the study. Participants were instructed about the experimental procedure and provided their informed consent. The experimental protocol complied with policies and principles of the Declaration of Helsinki and was approved by the Ethical Committee of the University of Milan (counsel 16/02/2021).

### Experimental protocol

**Fig. 1:**
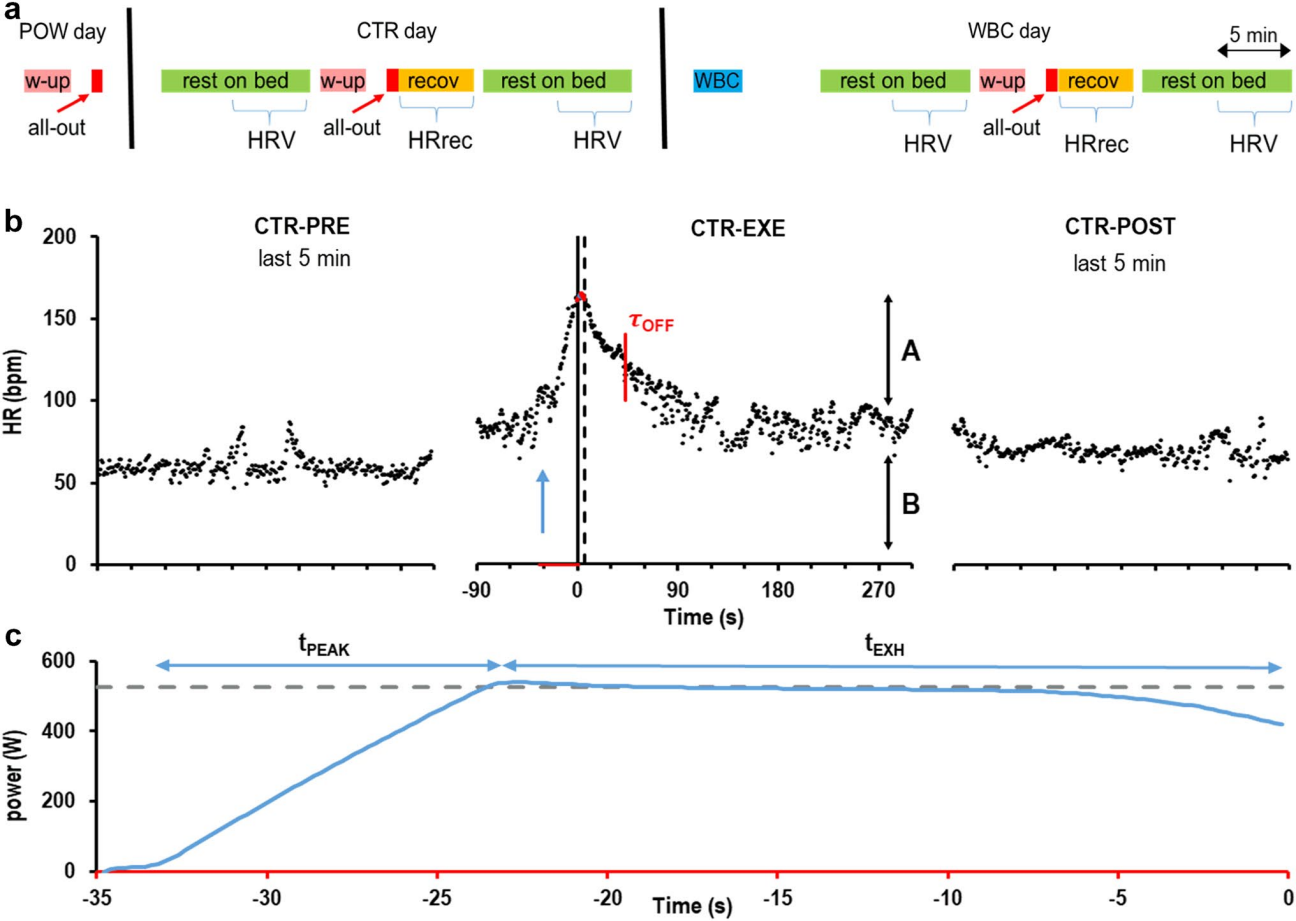
Experimental procedure. Timeline of the experiment is shown in panel **a** The three experimental days are described: maximal power measurement day (POW day), control evaluation day (CTR day) and post-cryotherapy evaluation day (WBC day). Black vertical bars mark the 3–5 days interval between experimental sessions. In color, the different experimental phases: 3 min warm-up, pink (w-up); maximal exercise, red (all-out); 10 min rest on bed, green; 5 min recovery, orange (recov); 3 min whole body cryotherapy, blue (WBC). Braces mark the time windows in which either the heart rate variability (HRV) or the heart rate recovery parameters (HRrec) were measured. Panel **b** illustrates the time course of the heart rate (HR) in a representative subject, recorded in CTR day. HR kinetics at rest before exercise (PRE, last 5 min), during exercise (EXE, maximal cycling from blue arrow to 0 s and recovery from 0 to 300 s), and at post-exercise rest (POST, last 5 min). In EXE, the thick vertical line represents the end of exercise while the dashed line indicates the onset of the HR exponential decay; thus, timing between these two lines marks t_DELAY_ (dots in red). The red vertical bar marks τ_OFF_, i.e. when HR decay was 63% completed. A and B symbolize the amplitude and the baseline of the mono-exponential decay, respectively. Panel ***c*** shows the time course of the instantaneous exerted power (blue trace) and the target power (grey dashed line) during exercise. The time axis corresponds to the red portion of the x-axis in panel ***b*** (from – 35 s to 0 s). As marked by the horizontal arrows, total exercise duration was splitted into the time needed to reach peak instantaneous power (t_PEAK_) and the time to exhaustion (t_EXH_)

Each subject was tested on three different days, interspersed by a range of 3–5 days (Fig. 1). On the first experimental day (maximal power measurement, POW day), the subject was instructed about the experimental procedure and familiarized with the cycloergometer (Lumed Eurobike 3200, Italy). When the subject felt confident about pedaling technique and posture, he/she was asked to warm-up for 3 min (60–70 rpm against 75 W load), followed by 5 min rest on the bike. The electromagnetic brake of the ergometer was then set so as to require 1000 W for performing about 100 rpm. An all-out exercise was then performed until exhaustion under verbal encouragement by the experimenter. The instantaneous power exerted by the subject was recorded by the cycloergometer; the maximal power was defined as the highest value that the could be maintained for 5 s. On the control day (CTR day), the subject was instrumented for EKG and breath recording (see below) and asked to rest for 10 min on a medical bed while recording cardiorespiratory activity (CTR-PRE). A second recording session was performed (CTR-EXE) in which: i) the subject got on the cycloergometer to warm up for 3 min and rest for 5 min, as in day 1; ii) he/she started the all-out exercise to reach the maximal power exerted on day 1; iii) continued pedaling until he/she could no more sustain 95% of the maximal power for 5 s (this event automatically triggered a loud beep signaling the subject to stop pedaling); iv) the subject seated still on the cycloergometer for 5 min, allowing HR to reach a post-exercise stationary value. Finally, a third session was recorded (CTR-POST) while the subject rested on the medical bed for 10 min. On the WBC day, after wearing thick wool gloves and socks, the subjects underwent cryotherapy in a cryosauna (CryoniqQ Cryo XC, Slovenia, 3 min in – 150 °C atmosphere) that excluded the head and neck. After WBC, they returned at room temperature, acclimated for 5 min and then repeated the experimental procedure of CTR day, leading to three new recording sessions (WBC-PRE, WBC-EXE, WBC-POST).

### Data acquisition

A standard three-lead EKG chest placement was used by positioning the active electrodes on both the acromions and the left iliac crest. Ground electrode was on the right iliac crest. A piezoelectric respiratory belt (PNG 156, Giunta Erasmo SAS, Italy) was placed on the chest at the level of the fifth intercostal space. Conventional Lead-II and Lead-III recordings were amplified by a portable EKG machine (Cardioline Delta 1 Plus, USA) while the respiratory signal was conditioned by a portable amplifier (gain 5 k, lowpass 100 Hz, Digitimer D440-4, England) before being acquired on a PC through an A/D board (500 Hz, 12 bits, National Instruments DaqCard 700). The same PC simultaneously communicated with the cycloergometer by RS232 to gather the cycling speed and exerted power values, as well as to control the target power, with a 5 Hz update rate.

### Measurements at rest

According to general practice reported in the literature, HRV was evaluated at rest on the bed during the last 5 min of the PRE and POST sessions, so as to grant stationarity of the R-R signal (cfr. Shaffer and Ginsberg 2017). In the same period, we also measured average breathing and heart rate (BR and HR, respectively). R-R intervals were pre-processed to exclude those falling outside ± 2 SD of the mean R-R duration. Then HRV was evaluated in the time and frequency domains: the time-domain parameter RMSSD was directly extracted from the R-R time sequence (Task Force of the European Society of Cardiology 1996) while the R-R sequence was resampled at 10 Hz, with spline interpolation, before applying the frequency domain analysis. The RMS Power Spectral Density was extracted from the resampled sequence by the Least-Squares autoregressive method, setting the order parameter to the value that minimized the Akaike information criterion, by a custom MATLAB routine (Task Force of the European Society of Cardiology ﻿^1996^). Mean model order (± SD) was 78 ± 20 in CTR-PRE, 96 ± 36 in CTR-POST, 80 ± 27 in WBC-PRE and 102 ± 38 in WBC-POST.

The two valleys in the power spectrum separating the very low frequency components (below approximately 0.04 Hz) from the low frequency peak (LF, about 0.04 to 0.15 Hz) and the LF peak from the high frequency one (HF, about 0.15 to 0.4 Hz) were visually identified. The upper limit of the HF peak was visually identified too, either as the valley separating HF from weak components at higher frequencies or, if no such valley was present, as the frequency at which the Power Spectral Density fell below 1% of HF peak value. The power in LF and HF bands was then evaluated as the area under the respective spectral peaks and expressed in ms^2^. Moreover, a quantitatively standardized index of the sympathovagal balance, before and after exercise and in CTR and WBC conditions, was obtained by dividing LF power by the so-called *total power* (LF power + HF power). The resulting parameter, LFnu, ranges from 0 to 100%; thus, these two limits represent the highest possible shifts of the sympathovagal balance toward vagal *vs.* sympathetic predominance, respectively. For completeness, we also calculated the LF/HF ratio that represents another well accepted index of sympathovagal balance.

### Exercise parameters

The ergometer target power in CTR and WBC days was set at the value of the maximal power reached by each individual in the POW day. In each trial we measured the time employed by the subject to reach the peak power output (t_PEAK_) and the time between such moment and the subject exhaustion (t_EXH_), signaled by the stop beep generated by the instrumentation. Average heart rate was evaluated during the last minute before starting the all-out exercise (HR_BEFORE_), i.e. at rest while sitting on the bike, and immediately after the end of exercise (HR_MAX_). HR data during all-out pedaling was not analyzed because of the many artifacts due to movement and respiration.

### Post-exercise HR decay parameters

The CTR-EXE and WBC-EXE recording sessions allowed us to estimate the time (t_DELAY_) for which the HR plateaus between the exercise cessation and the onset of the decay, as well as the time constant (τ_OFF_) of the exponential HR decay. In this aim, R-R intervals (in ms) from the end of the exercise to the end of EXE recordings were converted into instantaneous HR values (bpm) and fitted with a delayed exponential decay:

$$\left\{\begin{array}{cc}{\varvec{t}}\le {{\varvec{t}}}_{{\varvec{D}}{\varvec{E}}{\varvec{L}}{\varvec{A}}{\varvec{Y}}}& {\varvec{H}}{\varvec{R}}={\varvec{B}}+{\varvec{A}}\boldsymbol{ }\boldsymbol{ }\boldsymbol{ }\boldsymbol{ }\boldsymbol{ }\boldsymbol{ }\boldsymbol{ }\boldsymbol{ }\boldsymbol{ }\boldsymbol{ }\boldsymbol{ }\boldsymbol{ }\boldsymbol{ }\boldsymbol{ }\boldsymbol{ }\boldsymbol{ }\boldsymbol{ }\boldsymbol{ }\boldsymbol{ }\\ {\varvec{t}}>{{\varvec{t}}}_{{\varvec{D}}{\varvec{E}}{\varvec{L}}{\varvec{A}}{\varvec{Y}}}& {\varvec{H}}{\varvec{R}}={\varvec{B}}+{\varvec{A}}\boldsymbol{ }{{\varvec{e}}}^{\frac{{{\varvec{t}}}_{{\varvec{D}}{\varvec{E}}{\varvec{L}}{\varvec{A}}{\varvec{Y}}}-{\varvec{t}}}{{{\varvec{\tau}}}_{{\varvec{O}}{\varvec{F}}{\varvec{F}}}}}\end{array}\right.$$

where *t* is the time (in seconds) from the exercise cessation, *B* is the value (in bpm) at which HR settles at the end of the decay and *A* is the amplitude (in bpm) of the mono-exponential decay (Fig. 1B). To evaluate the time constant of the initial HR decay, we also calculated τ_30_, which expresses the rapid vagal reactivation that follows exercise (Imai et al. 1994). This was achieved by the same semi-logarithmic regression approach used by Imai et al. (1994), after subtracting from each HR value the final resting value (*B*) extracted from the exponential fitting procedure. Finally, average HR was measured during the last minute of the sitting recovery period (HR_AFTER_).

### Statistics

RMSSD, LF and HF power data, as well as the LF/HF ratio were log-transformed, to account for their lognormal distribution (Ziegler et al. 1999; Kim et al. 2009). Thereafter, Shapiro–Wilk tests certified the normal distribution of logRMSSD, logLF, logHF and logLF/HF, as well as of the other measurements. Separate two-way repeated-measures ANOVA, with factors *treatment* (CTR vs. WBC) and *exercise* (PRE vs. POST), could then be applied on logRMSSD, logLF, logHF, logLF/HF, LFnu, HR and BR data. Another two-way repeated-measures ANOVA was applied on HR data recorded in sitting position before, at the end and after exercise (CTR vs. WBC & HR_BEFORE_ vs. HR_MAX_ vs. HR_AFTER_). In addition, paired *t*-tests were used to compare t_PEAK_, t_EXH_, τ_OFF_, τ_30_ and t_DELAY_ values in CTR *vs.* WBC conditions. Statistical significance was granted at *p* < 0.05.

## Results

Experiments were conducted according to the timeline illustrated in Fig. 1a. The 28 subjects encompassed 2 women and 26 men. Mean anthropometric characteristics of the sample were: mean age 23.9 ± 3.8 years (SD), height 1.78 ± 0.09 m, body mass 74.1 ± 11.3 kg, BMI 23.23 ± 2.38 kg/m^2^. When resting supine on the bed the mean cardiac frequency was 66.0 ± 5.3 beats/min and the respiratory rate was 16.0 ± 2.4 acts/min. When pedaling on the cycloergometer in CTR and WBC days, the imposed target power was set to the maximal power measured in the POW day; in the different subjects, it ranged from 465 to 940 W (mean 725 ± 147 SD).

As shown in Table 1, a significant increase (14%) was found in t_EXH_ after WBC (t_27_ = 2.69, *p* = 0.0122). While no significant CTR *vs*. WBC changes were observed in t_PEAK_. For what concerns the HR recovery parameters, t_DELAY_ was significantly reduced by WBC (t_27_ = 3.30, *p* = 0.0027) while τ_OFF_ and τ_30_ values were unchanged. These data indicate that HR decays with the same slope but with an earlier onset after WBC. With regard to average HR (Table 2), the exercise clearly increased it while after WBC it was lower in all three measurements: before, at the end, and after exercise. Two-way ANOVA confirmed this finding with a significant effect of *exercise* (F_2,54_ = 1183, *p* < 0.0001) and of *treatment* (F_1,27_ = 4.824, *p* = 0.037), but no *interaction* (F_2,54_ = 0.89, *p* = 0.41).

**Table 1:** Parameters of the exercise and of the recovery phase

	t_PEAK_ (s)	t_EXH_ (s)	t_DELAY_ (s)	τ_30_ (s)	τ_OFF_ (s)
CTR	10.1 ± 0.1	13.2 ± 0.9	6.56 ± 0.47	72.6 ± 6.1	63.0 ± 3.3
WBC	10.2 ± 0.1	15.1 ± 0.8*	4.79 ± 0.47*	73.1 ± 6.0	65.0 ± 3.4

Time to peak power (t_PEAK_), time to exhaustion (t_EXH_), delay of the HR decay after exercise (t_DELAY_), time constant of the first 30 s of HR decay (τ_30_) and time constant of the overall recovery (τ_OFF_). Mean values ± SE, significant effects of whole body cryotherapy (WBC) with respect to control values (CTR) marked by *

**Table 2:** Average heart rate while sitting on the bike

	HR_BEFORE_ (beat/min)	HR_MAX_ (beat/min)	HR_AFTER_ (beat/min)
CTR	85.1 ± 2.7	175.1 ± 2.4	106.9 ± 3.1
WBC	80.6 ± 2.6*	173.7 ± 2.2*	104.2 ± 3.1*

Measurements were taken during the last minute before starting the all-out exercise (HR_BEFORE_), immediately after the end of exercise (HR_MAX_) and during the last minute of the sitting recovery period (HR_AFTER_). Mean values ± SE, significant effects of whole-body cryotherapy (WBC) with respect to control values (CTR) are marked by *

**Fig. 2:**
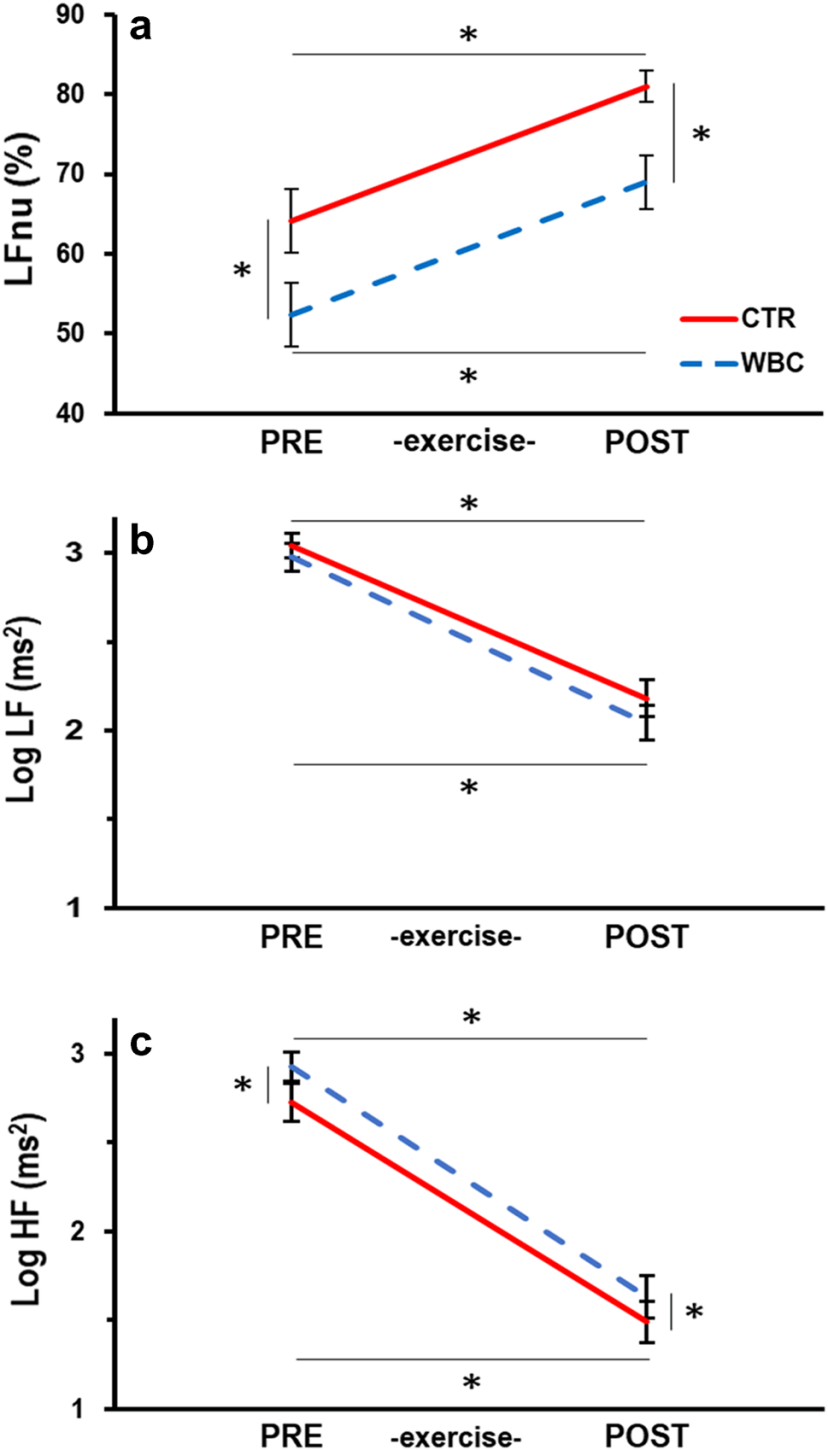
HRV analysis. LFnu, measured when lying supine on bed, is plotted in panel **a** All-out exercise significantly increased this parameter (POST vs. PRE) both in CTR condition (red line) and after WBC (blue). Instead, WBC significantly lowered LFnu, inducing a similar decrease both before and after exercise (CTR – WBC in PRE vs. CTR – WBC in POST). Panels **b** and **c** describe the effects of exercise and WBC on absolute power in the LF and HF frequency bands, respectively. Base-10 logarithmic units. While the exercise depressed both LF and HF power, WBC selectively enhanced HF power. Data expressed as mean ± standard error; significant differences marked by *

With regard to HRV, LFnu was significantly increased by the exercise (Fig. 2A, see also Arai et al. 1989). This was true both before WBC (LFnu changed from 64 to 81%, i.e. + 17%) and after the treatment (from 52 to 69%, i.e. + 17%). On the other side, WBC significantly depressed LFnu both before exercise (from 64 to 52%, i.e. – 12%) and after recovery (from 81 to 69%, i.e. – 12%). In other words, WBC was able to contrast about 70% of the effect produced by exercise. Repeated measures ANOVA confirmed these findings with main effects of exercise (F_1,27_ = 27.30, *p* < 0.0001) and treatment (F_1,27_ = 36.72, *p* < 0.0001) on LFnu, but no interaction (F_1,27_ = 0.004, *p* = 0.95). Exercise and treatment produced the same effects also on logLF/HF (treatment F_1,27_ = 39.27, *p* < 0.0001; exercise F_1,27_ = 26.81, *p* < 0.0001; interaction F_1,27_ = 0.02, *p* = 0.88). As expected, exercise also increased BR and HR. After recovery, both these rates were still significantly higher than before exercise (see Table 3), but values were unchanged after WBC. ANOVA indeed only found a main effect of exercise (BR: F_1,27_ = 43.04, *p* < 0.0001; HR: F_1,27_ = 408, *p* < 0.0001).

**Table 3:** Breath rate (BR), heart rate (HR) and indexes of sympathovagal balance, in both time (logRMSSD) and frequency domain (logLF/HF), measured when lying supine on bed, before exercise (PRE) and after full recovery (POST)

	BR (breath/min)		HR (beat/min)	
	PRE	POST	PRE	POST
CTR	16.0 ± 0.5	19.7 ± 0.6 §	66 ± 1	87 ± 2 §
WBC	16.5 ± 0.5	19.4 ± 0.6 §	64 ± 2	86 ± 2 §

	logRMSSD (ms)		logLF/HF	
	PRE	POST	PRE	POST
CTR	1.62 ± 0.05	0.97 ± 0.06 §	0.32 ± 0.09	0.69 ± 0.06 §
WBC	1.67 ± 0.04	0.98 ± 0.06 §	0.05 ± 0.08 *	0.41 ± 0.08 §*

Mean values ± SE, § marks significant changes between POST and PRE, while * marks significant changes produced by WBC vs. CTR

To ascertain whether the effects of exercise and WBC on LFnu stemmed from changes in LF power and/or in HF power, these data were analyzed (Fig, 2B, C). The exercise induced a significant decrease of both logLF (– 0.86 in CTR and – 0.94 in WBC), and logHF (– 1.23 in CTR and – 1.30 in WBC), whereas WBC selectively enhanced logHF (+ 0.20 before the exercise and + 0.14 after it). Statistical analysis confirmed a main effect of *exercise* on both logLF (F_1,27_ = 93.42, *p* < 0.0001) and logHF (F_1,27_ = 125.63, *p* < 0.0001) as well as a significant main effect of treatment only on logHF (logLF: F_1,27_ = 2.01, *p* = 0.17; logHF: F_1,27_ = 8.03, *p* = 0.0086), but no significant interactions (logLF F_1,27_ = 0.38, *p* = 0.54; logHF F_1,27_ = 0.22, *p* = 0.64). This confirmed that the WBC effect on LFnu should be mainly attributed to an enhancement in vagal drive, not modulated by exercise. With regard to the time-domain parameter logRMSSD (Table 3), which is also used to evaluate the vagal drive, we found that it was significantly reduced by exercise (F_1,27_ = 180.46, p =  < 0.0001) but not affected by the WBC treatment (F_1,27_ = 0.73, *p* = 0.40), nor by the *interaction* (F_1,27_ = 0.60, *p* = 0.44). Finally, the absence of WBC effects on BR and HR, which in turn may have affected HRV parameters, witnessed that the WBC-induced depression of LFnu and increase in HF actually represent changes in sympathovagal balance.

## Discussion

### Effect of WBC on exercise

Our data support the idea that WBC may be fruitful both for boosting exercise performance and for speeding up the post-exercise recovery. Indeed, we observed an increase in exercise duration (t_EXH_) and a shortening of the HR recovery (t_DELAY_) after a single session of WBC. In the present experiments, the pedaling power imposed by the cycloergometer after WBC was the same as that required in the CTR condition; thus, if participants had been requested to reach a higher power, they could have seemingly reached it, thanks to the boosting effect of WBC. Another clue of the enhancement in maximal power is the reduction of t_DELAY_ observed after the treatment. Indeed, many physiological markers, such as the blood lactic acid concentration, oxygen uptake, and ventilation, undergo a delayed exponential decay after strenuous exercise (Di Prampero et al. 1973; Margaria 1976; Bailey et al. 2018). Looking at the recovery curves described by those authors, one could observe that t_DELAY_ emerges as the exercise exceeds the aerobic threshold and gets longer as the load increases. Thus, the reduction of t_DELAY_ after WBC may indicate that the treatment increased the aerobic threshold and/or the maximal anaerobic load. Recalling the results by Otsuki et al. (2007) and Borresen and Lambert (2008), a WBC-induced increase in aerobic and/or anaerobic load should be accompanied to a decrease of τ_OFF._ However, this was not observed in our experiments. In this regard, it should be noted that Di Prampero et al. (1973) reported that τ_OFF_ was unchanged when comparing, in one and the same subject, the decay of O_2_ consumption after a supramaximal exercise to that observed after a maximal aerobic exercise. Thus, it is reasonable to expect that such a parameter does not change for all loads exceeding the aerobic threshold. In our experiments, the pedaling load was certainly above such a value, even after WBC, justifying that the treatment did not affect τ_OFF_.

Based on these arguments, future experiments should be envisaged to prove the boosting capability of WBC on maximal cycling power, as well as the time course of such effect. Moreover, since after WBC we observed a faster HR recovery despite the longer exercise duration, it would be also interesting to test whether an even faster recovery could be obtained when imposing the same exercise duration in CTR and after WBC. This would also point-out an underestimation of the effect of WBC on HR recovery in the present experiments. Such a result would add to the increase in stroke effectiveness reported in tennis players after repeated WBC sessions (Ziemann et al. 2012) and corroborate the perspective that WBC may enhance athlete preparation for competition (Partridge et al. 2019).

### Effect of WBC on sympathovagal balance

The current experiments also allowed to separately evaluate the effect of WBC and that of all-out exercise on sympathovagal balance, by measuring the quantitative index LFnu which derives from HRV analysis. It was shown that WBC decreased LFnu (about – 12%) while the exercise increased it (about + 17%). Surprisingly enough, the downward regulation induced by WBC largely compensated for the effect of exercise and was not modulated by it. Considering that LFnu depends on both the LF and HF components of HRV, we performed the analysis of absolute power in these two frequency bands and found that the WBC effect should be mainly attributed to an enhancement in HF power, which should be ascribed to an increase in vagal drive. This is in accordance with the study of Westerlund et al. (2006), in which healthy women were exposed to an acute session in a WBC chamber (2 min at – 110°, after 4 min of pre-acclimation). Indeed, these authors observed an increase in the HF component of HRV while resting in supine position and interpreted it as an increase in cardiac parasympathetic modulation. A brief comment is worth on the mechanisms underlying such effect: as indicated in the Introduction, the rapid cooling induces a potent skin vasoconstriction, which shifts the blood toward the central region of the body; the ensuing baroreceptor reflex would then be responsible for a depression of the sympathetic activity and an enhancement of the vagal drive. This is also consistent with the reduction of HR we found after WBC while seating on the cycloergometer before the exercise.

HRV is acknowledged as an indicator of the cardiac sympathetic and parasympathetic control (Task Force of the European Society of Cardiology 1996). In particular, the fraction of the total power in the low-frequency range (LF, 0.04–0.15 Hz) reflects both the sympathetic and the parasympathetic drive, while the fraction in the high-frequency range (HF, 0.15–0.4 Hz) unveils the parasympathetic drive through the vagus nerve. Despite Eckberg (1997) underlines that HF power is also strongly influenced by breathing, such parameter is at present considered the most representative index of vagal modulation (for a review, see La Rovere et al. 2020). This is also stressed by Cooke et al. (1999), who demonstrated a progressive decrease of HF power as a human subject was tilted from 0° up to 80°. Even considering that the mean values of HR and BR could have affected HRV parameters, it is worth noting that WBC did not significantly change neither of those rates when resting supine. This confirms that the decrease in LFnu and the increase in HF power are entirely due to the WBC effect. Theoretically, an increase in vagal tone should have been reflected in a decrease of HR. However, Shaffer and Ginsberg (2017) clearly explained that HF power represents vagal modulation of HR, not vagal tone (which in turn represents changes in average HR across conditions, e.g. from rest to exercise), thus the invariance of HR at rest in supine position does not contrast with our conclusion about sympathovagal modulation. Nevertheless, by looking at Table 2 it is apparent that WBC reduced more HR_BEFORE_ than HR_MAX_, actually supporting the enhancement of vagal tone too.

Considering the sympathovagal balance, some authors criticize that HRV analysis in the frequency domain may provide an unbiased index of such balance (Heathers 2012; Billman 2013), by sustaining various kinds of contaminations in LF and HF power measurements. Some authors even argued that the LF band reflects baroreflex activity in resting conditions and not cardiac sympathetic innervation (Goldstein et al. 2011; Rahman et al. 2011). Nevertheless, in the current literature, sympathovagal balance is commonly expressed by the ratio between the power in LF and HF bands, LF/HF, as well as by normalizing the power in each band to the sum of the powers in the two bands (LFnu, see methods, and HFnu). LF/HF has been reported to assess the balance between sympathetic and parasympathetic activity (Pagani et al. 1984, 1986; Malliani et al. 1991); some researchers have embraced this perspective (Tiller et al. 1996; Porges 2007; Shaffer et al. 2014). Moreover, LF/HF ratio has been shown to significantly change at exercise transitions (basal to exercise to recovery), accompanied by rapid changes in HR (Michael et al. 2017), and a low LF/HF ratio is associated with a better cardiac adaptation to daily life activities (Peçanha et al. 2014). Finally, the recovery time of LF/HF ratio after vigorous exercise seems to depend on individual cardiac respiratory fitness (Hautala et al. 2001). However, Burr (2007) highlighted that LF/HF, LFnu and HFnu are linked by a strict mathematical relation, so that all of them “should be considered equivalent carriers of information about sympathovagal balance”. In the present study, we focused on LFnu because of the strong linear correlation between this parameter and HR, reported by Bootsma et al. (1994) when tilting up a human subject from 0° to 60°. This supports the idea that LFnu proportionally increases when the sympathovagal balance shifts toward sympathetic predominance. In this way, the LFnu approach allowed an absolute quantification of the net effects of exercise and WBC. Nonetheless, we obtained the same results and significances of effects when repeating calculations with LF/HF, after logarithmic transformation to correct for its departure from the normal distribution (Ziegler et al. 1999; Kim et al. 2009; Schnell et al. 2013).

Another indication that WBC enhanced vagal drive comes from the analysis of t_DELAY_. Indeed, Storniolo et al. (2020) highlighted a relation between such delay and the subject’s aptitude to recover from the sympathetic overdrive induced by a strenuous exercise. According to that paper, a long t_DELAY_ is associated to a sympathetic activity that keeps high after exercise, thus the reduced t_DELAY_ we observed after WBC seems to be at all consistent with a faster vagal reactivation.

In parallel to the frequency-domain analysis, also time-domain parameters may reflect parasympathetic modulation. In this regard, we found that RMSSD, the time-domain analogue to HF power, was significantly reduced by exercise but unaffected by WBC. However, one should take into account that RMSSD not only includes those variability components which fall outside the frequency limits of the HF band, but it also weights the various components proportionally to their mean frequency. Thus, it is not unexpected that results obtained in the time-domain are less sensitive in evaluating the direction and magnitude of changes in vagal predominance than those in the frequency-domain, especially during short term recordings (see the review by Pumprla et al. 2002).

### Sympathovagal balance and exercise performance

Considering the parallel effects that WBC exerted on sympathovagal balance and exercise performance, it is tempting to draw a causal relationship between the vagal modulation enhancement and the performance boost. However, this is complicated by two issues. The first is that sympathovagal balance was measured while resting supine and exercise parameters while sitting on the cycloergometer, i.e. in two different postural conditions. In this regard, two facts should be considered: i) the direct proportionality between LFnu and the degree of tilting from supine to standing (Bootsma et al. 1994), and ii) the absence of interaction between the effects of WBC and exercise on LFnu (in fact, WBC reduced LFnu by the same quota before and after exercise). Thus, it is more than reasonable to expect that also the effect of posture added to that of WBC, without interaction (i.e. posture increased LFnu by the same quota, with and without WBC). This conclusion is also corroborated by the observation that the effect of WBC on LFnu (– 12%) was comparable to that exerted on t_EXH_ (+ 14%). All these considerations indicate that the change in posture should have not biased the relationship between vagal modulation enhancement and performance boost. The second issue is that other factors, linked to the strong temperature drop, may have influenced the exercise performance, e.g. the increase in venous return and stroke volume due to skin vasoconstriction, and/or direct metabolic effects on muscles. Present results do not allow dissecting these multiple contributions. Moreover, our results showed that the WBC effect on LFnu was present also after exercise. Therefore, it seems that WBC did not add an extra amount of energy to be spent in pedaling, but it likely improved the overall ability to produce external work. This is an additional clue in favor of the linkage between autonomic modulation and performance enhancement.

### Potential limitations of the study

It should be noted that in our experimental design the CTR and WBC days were not counterbalanced. In this regard, it is improbable that a single all-out effort may abruptly trigger a long lasting training effect. Actually, it is well known that performance improves only after repeated training sessions, lasting weeks and encompassing multiple exercise repetitions per day. The 3–5 days interval between the experimental sessions should then ensure that complete washout from previous exercise occurred. On the other side, since the duration of the WBC effect is still debated, it was applied on the last day to avoid any carryover effect. Another possible consequence of the lack of counterbalancing is that subjects underwent a “familiarization”, possibly leading to a repeated bout effect. Indeed, Gething et al. (2004), who studied the effects of respiratory muscle training on cycling, showed that when subjects perform three time trials, the second and the third would be longer than the first one. However, the same authors reported no changes in time to exhaustion between the second and third cycling trials. Since our CTR and WBC trials were actually the second and third ones, the first being the POW, Gething’s data indirectly suggest that the familiarization effect should not have biased any difference in cycling performance we observed between CTR and WBC.

Another possible limitation comes from the composition of our experimental sample, which contained 26 males and 2 females. According to Hammond et al. (2014), “the effects of sex and anthropometrics should be considered when designing WBC research”. These authors indeed reported that WBC induced greater cooling in female subjects than in males and the subjects with a higher adiposity cooled more than thinner individuals. In this regard, it should be noted that female subjects in Hammond’s sample were consistently fatter than males. For what concerns our sample, the two females had a BMI of 20.20 and 26.96 respectively, i.e. close to the two extremes of the range of male subjects (19.35 and 29.77), suggesting that female data did not biased the results. In any case, Westerlund et al. (2004) reported that “neither significant gender differences nor adaptation in blood pressures were found during WBC”. Finally, repeating the statistical analysis limited to our male participants confirmed the effects observed in the whole population.

## Data Availability

The datasets generated for this study are available on reasonable request to the corresponding author.
